# Audit of adherence to GI screening recommendations for Lynch Syndrome Patients

**DOI:** 10.1186/1897-4287-10-S2-A50

**Published:** 2012-04-12

**Authors:** Lucinda Hossack, Omid Zarghom, Gillian Mitchell, Mary Shanahan, Craig Lynch, Alexander Heriot, Alex Boussioutas

**Affiliations:** 1 Familial Cancer Centre, Peter MacCallum Cancer Centre, East Melbourne, Australia

## 

There is clear evidence documenting the morbidity and mortality benefit of regular colonoscopy surveillance in reducing the high risk of primary and metachronous colorectal cancers associated with Lynch Syndrome (LS) (Jarvinen et al. 2000). The Australian NHMRC guidelines (2005) recommend LS patients undergo a colonoscopy every 1-2 years (NHMRC 2005). At Peter Mac yearly colonoscopy screening is usually advised for LS patients.

The long term adherence rate to colonoscopy in LS patients as reported in the literature is between 60-88% (Stoffel et al 2010, Wagner et al 2005). Some factors have been associated with inadequate LS patient screening adherence including lack of sedation, inappropriate advice from managing doctors, financial costs, embarrassment and lack of patient time (Bleiker et al 2005). Whilst not studied, some of these factors may also contribute to a phenomenon known as ‘screening fatigue’, whereby after one or more normal screening procedures patients begin to attend less frequently for the recommended screening procedures.

At the Peter Mac patients and their managing doctors are given GI screening recommendations by the FCC when the patient receives their gene mutation results. However, it is unclear whether LS patients continue to follow the GI screening advice set out by an FCC over time and whether there is any evidence that ‘screening fatigue’ exists. Furthermore it is unknown whether differences regarding the frequency of colonoscopy offered/performed, adherence to colonoscopy or quality of endoscopy exist for patients who participate in a high risk GI management clinic versus those who are screened by endoscopists in generalist community based settings.

The FCC at Peter Mac undertook an audit of the GI screening practices of 74 confirmed Lynch Syndrome gene mutation carrier patients from 2006-2010. Of these patients 27 participate in a high risk GI management clinic where they receive their GI screening. The additional 47 patients have GI screening conducted through either private endosocopy clinics or at public hospitals.

This work will;

❖ Provide a clinical description of our population of LS patients

❖ Describe the rate of adherence to recommended GI screening overtime. This is assessed by comparing the screening practices undertaken with the recommendations provided by the FCC at the time of their gene test result. We will also present data about whether updates of screening recommendations by our FCC were acted upon by patients/their managing doctors.

❖ Assess for evidence of ‘screening fatigue’

❖ Compare rates of adherence to GI screening practices between patients attending GI high risk management clinics versus patients attending for GI screening in community settings

❖ Describe the overall colorectal polyp and GI cancer detection rate

❖ Use colorectal polyp detection also a measure of quality of colonoscopy

We will make some suggestions for further research particularly into interventions that may improve adherence to GI screening advice for a population of LS patients.

